# Medicinal Fungus *Antrodia cinnamomea* Inhibits Growth and Cancer Stem Cell Characteristics of Hepatocellular Carcinoma

**DOI:** 10.1155/2013/569737

**Published:** 2013-03-06

**Authors:** Yu-Ming Liu, Yu-Kuo Liu, Keng-Li Lan, Yu-Wei Lee, Tung-Hu Tsai, Yu-Jen Chen

**Affiliations:** ^1^Cancer Center, Taipei Veterans General Hospital, Taipei 11217, Taiwan; ^2^Institute of Traditional Medicine, School of Medicine, National Yang Ming University, Taipei 11221, Taiwan; ^3^Department of Chemical and Material Engineering, Chang Gung University, Kwei-Shan, Tao-Yuan 333, Taiwan; ^4^Department of Education and Research, Taipei City Hospital, Taipei 10341, Taiwan; ^5^Department of Radiation Oncology, Mackay Memorial Hospital, Taipei 10449, Taiwan

## Abstract

*Background*. *Antrodia cinnamomea* is an edible fungus commonly used in Asia as a well-known medicinal herb capable of treating drug intoxication and liver cancer. *Methods*. This study evaluated the anticancer activity of its biotechnological product, mycelial fermentation broth (*AC*-MFB) on hepatocellular carcinoma (HCC) by tetrazolium-based colorimetric assay *in vitro* and syngeneic Balb/c 1MEA.7R.1 tumor implantation model *in vivo*. Given that cancer stem cell characteristics, such as angiogenesis, invasiveness, and migration, are known to cause recurrence, we further evaluated the effect of *AC*-MFB on cellular viability inhibition of HCC cells, angiogenic activity and migration of endothelial cells, and the release of proangiogenic factors from HCC cells. *Results*. We found that *AC*-MFB markedly inhibited the growth of HCC without hepatic enzyme abnormality. This anti-HCC activity was validated by growth-inhibitory effects on both cultured murine 1MEA.7R.1 and human HA22T/VGH HCC cells. For cancer stem cell characteristics, *AC*-MFB inhibited the cellular viability, migration, and tube formation activity of EA. hy926 and SVEC4-10 endothelial cells. Production of extracellular vascular endothelial growth factor and intracellular hypoxia-inducible factor-1 alpha from HCC cells was suppressed by *AC*-MFB. *Conclusion*. *Antrodia cinnamomea* could inhibit the growth and cancer stem cell characteristics of HCC cells.

## 1. Background

*Antrodia cinnamomea,* also known as niu-chang-chih, *Taiwanofungus camphorate* or *Antrodia camphorata*, is a fungus indigenous to Taiwan which grows on decayed *Cinnamomum kanehirae* [1–3]. In folk medicine, it is well known for its antidotal and antitumoral functions as it has been traditionally used to treat toxicities caused by food, alcohol, and drugs as well as diarrhea, abdominal pain, hypertension, itchy skin, and tumors [4, 5]. 

*A. cinnamomea* has been shown to exhibit anticancer properties [6–9] with many studies aimed at exploring the exact bioactive compounds [10–14]. For example, Chen et al. reported that the oral administration of *A. cinnamomea *fruiting bodies significantly increased the life span of ATCC BNL IMEA.7R.1 hepatoma-bearing mice [15]. It has also been reported that polysaccharides from *A. cinnamomea* mycelial extract could inhibit angiogenic activities in endothelial cells (ECs) [16–18]. 

To avoid contamination in the wild source and to expand the production of this fungus for food use, *A. cinnamomea* mycelial fermentation broth (*AC*-MFB) produced using biotechnology has been developed by our group and others. Although *AC-*MFB is not used as natural form, it could be regarded as a modern formulation of this herbal drug. However, the anticancer activity and safety of these biotechnological products have not been extensively examined. 

We previously purified a series of compounds from the fruiting body of *A. cinnamomea,* including 24-methylenelanosta-7,9(11)-diene-3b,15adiol-21-oic acid (designated herein as MMH01), which possesses cytotoxicity against human leukemia and pancreatic cancer cells [19]. In the present study, we investigated the anticancer effect and safety of *AC*-MFB with a syngeneic hepatocellular carcinoma (HCC) implantation model. The effect of *AC*-MFB against cancer stem cell characteristics in HCC and endothelial cells was also evaluated. 

## 2. Methods

### 2.1. Plants Materials, Fermentation, and Characterization

*A*. *cinnamomea*, strain B137, identified by fungi specialist Dr. T. T. Chang (Taiwan Forestry Research Institute, Taipei, Taiwan), was maintained on potato dextrose agar pasteurized Petri dishes and transferred to a fresh medium at 1-month intervals. Mycelial agar discs (8 pieces, 0.5 cm each) were obtained by a sterilized tip and used as the inoculum in a shake flask preculture. The preculture medium (LM-B) consisted of the following components (mg/mL): glucose 30, sucrose 20, yeast extract 15, peptone 13, MgSO_4_ 0.3, KH_2_PO_4_ 0.3, and K_2_HPO_4_ 0.3 with an initial condition of pH 4.0. For the preculture, a 200 mL medium was prepared in a 500 mL flask and inoculated, followed by a 7-day incubation at 28°C on a rotary shaker (100 rpm). For a series of experiments, a 100 mL medium (LM-B) was prepared in a 500 mL flask and inoculated with mycelium suspension (6%) from the preculture broth, followed by a 14-day incubation at 28°C on a rotary shaker (100 rpm). The fermentation product was then harvested and poured through a nonwoven fabric on a 20-mesh sieve to separate the deep-red fermented culture broth and the mycelia, followed by centrifugation at 3000 g for 10 min and then by passage through a filter of 0.22 *μ*m pore size. The culture broth (1.0 L) was then concentrated into a colloid (47.6 mg) under vacuum and stored at −30°C before the analysis. The* AC*-MFB colloid was redissolved and directly taken into the study without further extraction. The polysaccharide components in* AC*-MFB were used in quantity analysis for standardization in this study. The phenol-sulfuric acid assay, a colorimetric method, was employed to determine the total concentration of carbohydrates present in *AC*-MFB [20]. 

### 2.2. *In Vivo* Tumorigenesis and Hepatic Toxicity Assay in Mice

 A subcutaneous allogenic mice animal model was used for tumorigenesis assay. Specific pathogen-free male BALB/c mice (4 weeks old, 25–28 gm) were obtained from the National Laboratory Animal Center (Taipei, Taiwan) and were maintained in pathogen-free conditions. They were kept in our animal facility for at least two weeks before use. All of the mice were used at the age of 6–8 weeks. All animals were cared for following the *Guide for the Care and Use of Laboratory Animals* (NIH publication no. 85-23, revised in 1985). The mice were subcutaneously injected with 1 × 10^5^ 1MEA.7R.1 HCC cells over the flank. The mice were fed orally either with 50 mg/mL* AC*-MFB 200 *μ*L or with a vehicle of normal saline 200 *μ*L (*N* = 6 for each group) for 28 days. The tumor size of each mouse was measured by a caliper and calculated by the formula: *L* × *W* ^2^/2, where *W* was the shortest dimension and *L* was the longest dimension in centimeters. The tumor size was estimated every 2 to 3 days after the tumor size increased to 0.1 mL in size. Tumor growth curves were plotted as the mean tumor volume relative to the treatment group. 

Hepatic enzyme alanine aminotransferase (ALT) is found primarily in the cytoplasm of hepatic cells which catalyses gluconeogenesis from noncarbohydrate sources and is an important marker for liver injury [21, 22]. Elevation of the serum concentrations of this marker implied disruption of plasma membrane integrity, which eventually led to leakage of the enzymes into the blood circulation [23]. ALT was used as a biochemical marker for hepatic damage assay in this study. Animals were fed with different doses of *AC*-MFB by gastric tube for each condition for 28 days (Table 1); each group had 6 mice. Blood samples were collected for the assays of ALT on the first day as a control and on the third day and the 28th day for acute and chronic hepatic toxicity assays, respectively. Serum activities of ALT were determined using the colorimetric method [24].

The protocol used in this study was approved by the Institutional Animal Care and Use Committee, Chang Gung University, Taiwan (IACUC approval no. CGU10-143).

### 2.3. *In Vitro* Cytotoxicity Assays

The* in vitro* cytotoxicities of the *AC*-MFB on both HCC cells and ECs were examined using a modified MTT assay [25]. The human ECs EA. hy926 (a gift from Dr. Cora-Jean S. Edgell, Pathology Department, University of North Carolina), human HCC cell HA22T/VGH [26], and the BALB/c murine BNL 1MEA.7R.1 (BNL) HCC cells were grown in DMEM, containing 584 mg/mL L-glutamine and 10% FBS. The murine SV40-transformed mouse ECs (SVEC4-10, ATCC no. CRL-2181) were maintained in DMEM with 10% FBS. 

### 2.4. *In Vitro* Antiangiogenic Assays

The antiangiogenic activity was investigated by the capability of endothelial cell migration and tube formation on Matrigel *in vitro*.

Endothelial cell migration was determined by means of a wound-healing assay. ECs were seeded into 6-well tissue culture dishes and cultured in medium containing 10% FBS to confluent cell monolayers, which were then carefully wounded using sterile 200 *μ*L pipette tips with all cell debris removed by phosphate buffered saline (PBS). The cells were then incubated for another 24 h in medium with or without *AC*-MFB treatment. The cells that had migrated across the edge of the wound were photo recorded under an inverted phase-contrast microscope (Nikon, Tokyo, Japan), and the cellular migration was determined by measuring the widths of the wounds. The differences in the width of the wounds were measured with Adobe Photoshop version 5.5 (Adobe Systems Incorporated, USA). Each data point was compared with its own control present in the control dish at 24 h and interpreted as a percentage of wound migration inhibition. 

Matrigel (BD BioCoat Angiogenesis System, Franklin Lakes, NJ, USA) was employed to promote the differentiation of ECs into capillary tube-like structures. A total of 75 *μ*L of thawed Matrigel was added to 96-well tissue culture plates, followed by incubation for 60 min at 37°C to allow polymerization. Then, 4 × 10^4^ quiescent ECs were seeded on the gels in medium supplemented without FBS in the presence or absence of *AC*-MFB, followed by incubation at 37°C in 5% CO_2_ for 12 h. Tube formation was examined by an inverted phase-contrast microscope (Nikon, Tokyo, Japan). The number of the tubes was counted with Adobe Photoshop version 5.5 (Adobe Systems Incorporated, USA). Each data point was compared with its own control (without *AC*-MFB) at 12 h and interpreted as a percentage of tube formation inhibition. 

### 2.5. Inhibition of Proangiogenic Factors from Hepatic Cancer Cells

Secretion of VEGF and HIF-1*α* by HCCs was stimulated by 2 h incubation with 200 *μ*M desferrioxamine (DFO) (Sigma, St. Louis, MO, USA) to mimic hypoxia. VEGF levels of HA22T and 1MEA.7R.1 HCCs were measured by Human VEGF ELISA kit no. DY293B and Mouse VEGF ELISA no. DY493, respectively. The HIF-1*α* was measured using a solid-phase enzyme immunoassay (“sandwich ELISA,” NOVUS and NeoMarkers, Inc.). Each measurement was done in triplicate, and the data were calculated and normalized to total protein in the culture media. 

### 2.6. Statistical Analysis

Data from three separate experiments were expressed as a mean ± standard deviation (SD). Statistical comparisons of the results were made using analysis of variance [27] as indicated. Significant differences (*P* < 0.05) between the means of the two test groups were analyzed by Tukey's test.

## 3. Results

### 3.1. Characterization and Stability of* AC*-MFB

Although the bioactive ingredients of *AC*-MFB remain to be determined, the amount of polysaccharides in a concentrated* AC*-MFB colloid was used as the standard of quality control to characterize each batch of product. The phenol-sulfuric acid method showed that there were 0.341 mg polysaccharides in 47.6 mg *AC*-MFB colloid from 1.0 L fermentation broth. The polysaccharides shared 0.7% weight in the concentrated cultured broth colloid. There was no significant change in the amount of polysaccharides in* AC*-MFB throughout the 12 months of storage, indicating it is a product with long-term stability.

### 3.2. *AC-*MFB Inhibited Tumor Growth in Mice without Hepatic Enzyme Abnormality

**Figure 1:**
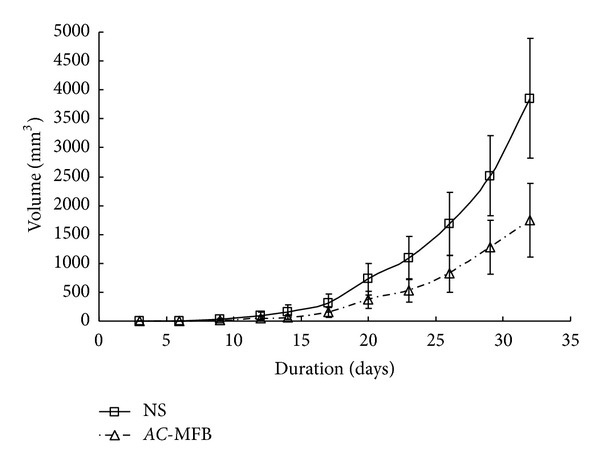
Antitumor effect of *AC*-MFB on BALB/c liver cancer. Effect of *AC*-MFB on growth of 1MEA.7R.1 liver cancer cells in BALB/c mice. Mice were treated with oral *AC*-MFB (50 mg/mL) or normal saline for 28 days. *P < 0.05.

In BALB/c mice bearing 1MEA.7R.1 liver cancer cells, oral intake of 200 *μ*L *AC*-MFB (50 mg/mL) for 28 days significantly inhibited the tumor growth in comparison with the vehicle control (*P* < 0.05) (Figure 1).

Mice serum ALT levels at 3 days and 28 days after treatment were examined for estimation of liver toxicity. As shown in Table 1, there was no statistically significant difference between serum ALT levels of *AC*-MFB and control groups after 3 days and 28 days of *AC*-MFB oral intake.

### 3.3. *AC*-MFB Inhibited Cellular Viability of HCC Cells and ECs

**Figure 2:**
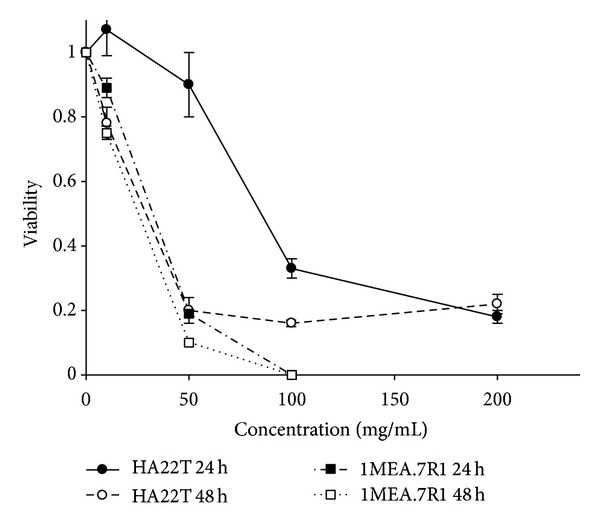
Cytotoxicity of *AC*-MFB on hepatocellular carcinoma cell. Effect of *AC*-MFB on cellular viability of HA22T and 1MEA.7R.1 HCC cells by MTT assay. Data from three separate experiments were expressed as percentages of untreated cells after 24 and 48 h.

**Figure 3:**
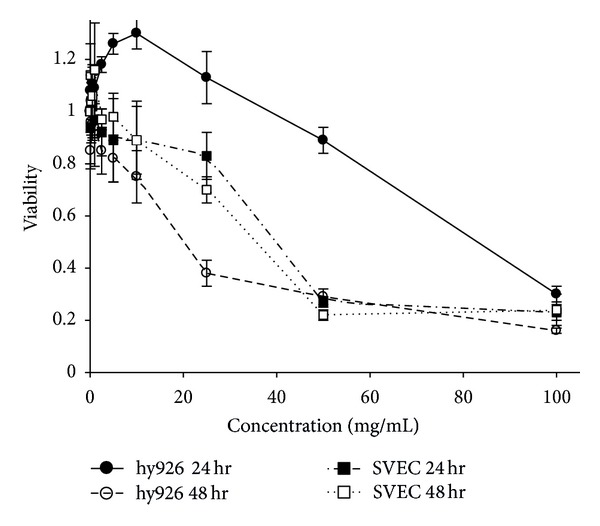
Cytotoxicity of *AC*-MFB on endothelial cell. Effect of *AC*-MFB on cellular viability of EA. hy926 and SVEC4-10 ECs by MTT assay. Data from three separate experiments were expressed as percentages of untreated cells after 24 and 48 h.

As shown in Figures 2 and 3, *AC*-MFB inhibited the viability of HCC cells (human HA22T/VGH, murine 1MEA.7R.1) and ECs (EA. hy926, SVEC4-10) in both dose-dependent and time-dependent parameters.

### 3.4. *AC*-MFB Inhibited Endothelial Cell Migration and Tube Formation Activity

**Figure 4:**
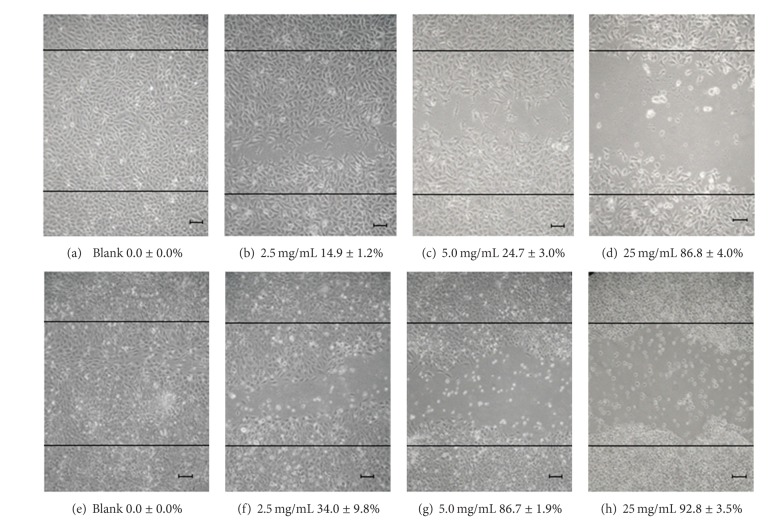
Migration inhibition of *AC*-MFB on endothelial cell. Effect of *AC*-MFB on migration of EA. hy926 ((a)–(d)) and SVEC4-10 ((e)–(h)) ECs by *in vitro* wound-healing migration assay. Data from three separate experiments were expressed as mean inhibition percentages compared with untreated cells after 24 h treatment.

**Figure 5:**
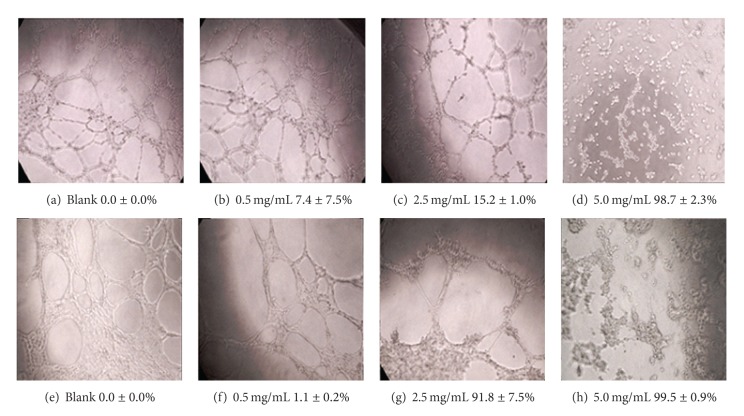
Tube formation inhibition of *AC*-MFB on endothelial cell. Effect of *AC*-MFB on tube formation activity of EA. hy926 ((a)–(d)) and SVEC4-10 ((e)–(h)) ECs. Data from three separate experiments were expressed as mean inhibition percentages compared with untreated cells after 12 h treatment.

The migration and tube formation activity of ECs are important *in vitro* endpoints for angiogenesis. As demonstrated in Figure 4, *AC*-MFB profoundly inhibited the migration of EA. hy926 and SVEC4-10 cells in a concentration-dependent manner. The IC_50_ values of migration inhibition of EA. hy926 and SVEC4-10 were 8.8 ± 1.6 mg/mL and 3.0 ± 0.3 mg/mL, respectively. The tube formation activity in these two endothelial cell lines was also inhibited by treatment using *AC*-MFB with IC_50_ ranging from 1.4 to 3.1 mg/mL (Figure 5). 

### 3.5. *AC*-MFB Suppressed Production of VEGF and Downregulated Intracellular HIF-1*α* Levels in HA22T/VGH and 1MEA.7R.1 Cell Lines

**Figure 6:**
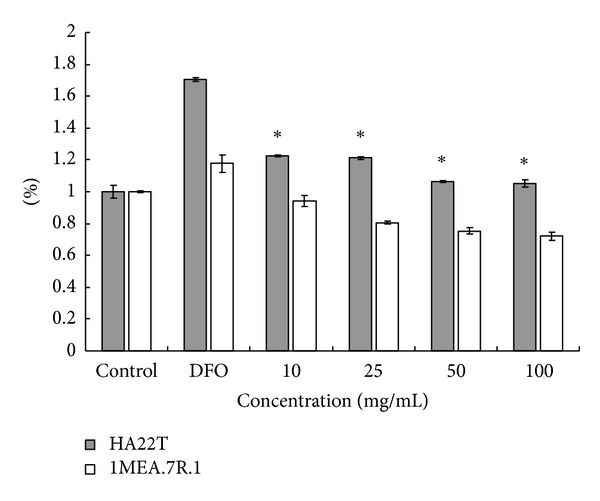
*AC*-MFB inhibits VEGF from hepatocellular cancer cell. Effect of various concentrations of *AC*-MFB on extracellular VEGF levels in culture of HA22T and 1MEA.7R.1 HCC cells. Cells were pretreated with 200 *μ*M DFO for 2 h followed by *AC*-MFB for 6 h. All the data are normalized to the control group (*AC*-MFB 0.0 mg/mL). Data from three independent experiments in triplicate were expressed as mean ± standard error of mean; *P < 0.05 versus 200 *μ*M DFO.

Given that VEGF plays a critical role in angiogenesis, we investigated whether* AC*-MFB could modulate the secretion of VEGF from HCC cells. As shown in Figure 6, *AC*-MFB treatment suppressed the DFO-stimulated VEGF secretion in both HCC cell lines, but only the difference in HA22T/VGH cells reached statistical significance (*P* < 0.05). 

**Figure 7:**
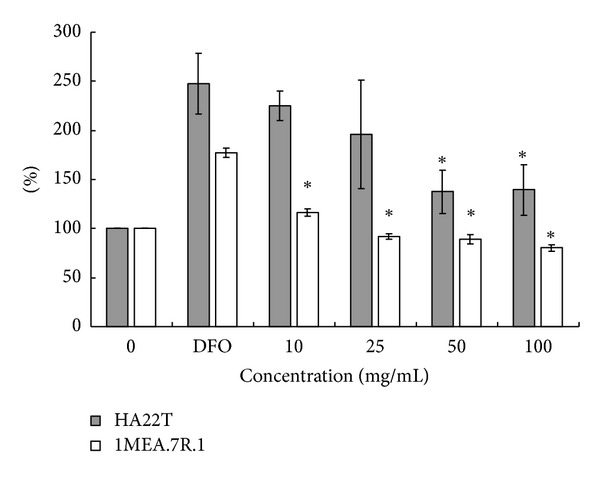
*AC*-MFB inhibits HIF-1*α* from hepatocellular cancer cell. Effect of various concentrations of *AC*-MFB on intracellular HIF-1*α* levels in culture of HA22T and 1MEA.7R.1 HCC cells. Cells were pretreated with 200 *μ*M DFO for 2 h followed by *AC*-MFB for 2 h. All the data were normalized to the control group (*AC*-MFB 0 mg/mL). Data from three independent experiments in triplicate were expressed as mean ± standard error of mean; *P < 0.05 versus 200 *μ*M DFO.

Hypoxia-inducible factor-1*α* (HIF-1*α*) is a protein that regulates gene transcription through binding to hypoxia response elements, resulting in upregulation of VEGF and thereby enhancing angiogenesis. *AC*-MFB treatment significantly downregulated the DFO-stimulated intracellular HIF-1*α* levels in both HCC cell lines (*P* < 0.05) (Figure 7). 

## 4. Discussion 

In this study, we examined the *in vivo* activity of *AC*-MFB first. Since the animal model showed it was effective without major toxicity, we next evaluated the *in vitro* effect of *AC*-MFB on both HCC cells and endothelial cells to explore possible targets. Based on the antiangiogenesis activity of *AC*-MFB, we further assessed the modulation of angiogenic factors by *AC*-MFB.

The results showed that *AC*-MFB could inhibit the viability of HCC cells (human HA22T/VGH, murine 1MEA.7R.1) and ECs (EA. hy926, SVEC4-10) in both dose-dependent and time-dependent parameters *in vitro* (Figures 1 and 2). The *AC*-MFB could also inhibit the migration and tube formation activities in both ECs (Figures 4 and 5). *In vivo*, oral intake of *AC*-MFB inhibited the tumor growth in BALB/c mice bearing 1MEA.7R.1 liver cancer cells without hepatic enzyme abnormality (Figure 3, Table 1). In bimolecular activity, *AC*-MFB could suppress the secretion of VEGF and downregulate the HIF-1*α* level (Figures 6 and 7). 

The *A. cinnamomea* mycelial fermentation broth, a biotechnological product serving as a medicinal herb, could inhibit the growth of hepatocellular carcinoma* in vivo* without liver toxicity. Further investigation to clarify the antitumoral mechanisms revealed an inhibitory activity on angiogenesis and the release of related proangiogenic factors VEGF and HIF-1*α*, indicative of inhibitory activity related to cancer stem cell characters.

Natural *A. cinnamomea *is very rare and slowly growing. Moreover, the host wood, *Cinnamomum kanehirae,* is a local species that is getting scarce and difficult to find in the forest. Therefore, the extracts of the fruiting body are difficult to obtain for use as a medicinal herb. Through biotechnological production in a fermentation broth and the examination of its bioactivity against HCC, a scientific basis for* AC*-MFB to serve as a medicinal herb in adjunct cancer treatment was found. 

Hepatocellular carcinoma is one of the most frequently occurring malignancies with poor prognosis in Taiwan [28]. *A. cinnamomea* has attracted great attention due to its hepatoprotective effect and antitumoral activity against HCC [4, 7, 9]. Experimental and clinical data had indicated that HCC tumor progression is associated with angiogenesis and that an increase in microvascular density is associated with a poor prognosis [29]. Given that HCC is characterized by hypervascularity [30], antiangiogenesis might be one of the primary treatment approaches [31]. 

Our study showed that not only did* AC*-MFB have antitumoral activity against HCC cells *in vitro*, it also delayed 1MEA.7R.1 HCC growth without hepatic enzyme abnormality* in vivo*. In other words, *AC*-MFB might be a potential therapeutic agent or an adjuvant regimen for treatment of HCC.

Furthermore, *AC*-MFB inhibited the cellular events related to cancer stem cells, such as angiogenesis, migration, and HIF-*α* expression. For antiangiogenesis, our study also showed that *AC*-MFB could inhibit the cellular viability, migration, and tube formation of ECs and decreased both the extracellular VEGF and the intracellular HIF-1*α* levels of HCC cells under hypoxia. These findings suggest that the antiangiogenic activity of *AC*-MFB might involve inhibition of HIF-1*α* transcription and downregulation of VEGF expression in HCC cells. Such claims could be supported by previous investigations. For example, Yang reported that fractionated polysaccharides from *A. cinnamomea* mycelia, with molecular weight (MW) > 100 kDa, are antiangiogenic *in vitro *and *ex vivo*, and the effects are likely through immunomodulation [17]. Cheng et al. not only reported that polysaccharides extracted from fermented *A. cinnamomea* mycelia could inhibit VEGF receptor phosphorylation and angiopoietin-2 protein expression but also found the chemical structure of the polysaccharide [16, 18]. Shih et al. also reported that extracted polysaccharides from the fruiting body of *A. cinnamomea* with a molecular weight between 2693 and 2876 kDa had a critical inhibitory effect on tube formation without cytotoxicity [32]. Polysaccharides were also proved to elicit an antitumoral effect by promoting a Th1-dominant state and killer activities [6]. The polysaccharides only shared 0.7% weight of our concentrated cultured broth colloid; soother lanostane-type components like MMH01 isolated from *A. cinnamomea* could have anticancer activity [33]. Taken together, polysaccharides may not be the major bioactive component in *AC*-MFB. Instead, the amount of exopolysaccharide could serve as a good standardization index for each batch of fermented products. 

Our study is the first one demonstrating the antiangiogenic activity of *A. cinnamomea* mycelial fermentation broth, not from extracts of fruiting body. It indicates that medicinal herbal products of *A. cinnamomea* may possess the original bioactivity against HCC and angiogenesis. 

## 5. Conclusion

The *A. cinnamomea* mycelial fermentation broth, a biotechnological product serving as a medicinal herb, could inhibit growth of hepatocellular carcinoma* in vivo*. Furthermore, *AC*-MFB could inhibit the cellular events related to cancer stem cells, such as angiogenesis, migration, and HIF-*α* expression in HCC. 

## Figures and Tables

**Table 1:** Effect of *AC*-MFB on ALT levels in mice.

Treatment group	Dose (g/Kg BW)	Day 0	Day 3	Day 28
Normal saline 200 *μ*L	0.0	75 ± 20	96 ± 62	60 ± 2
50 mg/mL 200 *μ*L	0.4	49 ± 12	48 ± 11	44 ± 4
100 mg/mL 200 *μ*L	0.8	111 ± 52	60 ± 12	126 ± 42
250 mg/mL 200 *μ*L	2.0	78 ± 42	44 ± 10	44 ± 2
500 mg/mL 200 *μ*L	4.0	73 ± 36	93 ± 42	77 ± 22
500 mg/mL 300 *μ*L	6.0	130 ± 95	56 ± 15	160 ± 79

All values represent mean ± SD. Normal value of mouse ALT: 28–132 U/L.

*N* = 6 for each group.

## Abbreviations

*AC*-MFB: *An* *tr* *od* *ia* *cinnamomea* mycelial fermentation broth
HCC: Hepatocellular carcinoma
ECs: Endothelial cells
MTT: Modified tetrazolium-based colorimetric assay (3-(4,5-dimethylthiazol)-2,5-diphenyl tetrazolium bromide assay
VEGF: Vascular endothelial growth factor
HIF-1*α*: Hypoxia-inducible factor-1*α*
ALT: Alanine aminotransferase.
